# Oral contraceptives, reproductive history and risk of colorectal cancer in the European Prospective Investigation into Cancer and Nutrition

**DOI:** 10.1038/sj.bjc.6605965

**Published:** 2010-11-02

**Authors:** K K Tsilidis, N E Allen, T J Key, K Bakken, E Lund, F Berrino, A Fournier, A Olsen, A Tjønneland, K Overvad, M-C Boutron-Ruault, F Clavel-Chapelon, G Byrnes, V Chajes, S Rinaldi, J Chang-Claude, R Kaaks, M Bergmann, H Boeing, Y Koumantaki, G Stasinopoulou, A Trichopoulou, D Palli, G Tagliabue, S Panico, R Tumino, P Vineis, H B Bueno-de-Mesquita, F J B van Duijnhoven, C H van Gils, P H M Peeters, L Rodríguez, C A González, M-J Sánchez, M-D Chirlaque, A Barricarte, M Dorronsoro, S Borgquist, J Manjer, B van Guelpen, G Hallmans, S A Rodwell, K-T Khaw, T Norat, D Romaguera, E Riboli

**Affiliations:** 1 Cancer Epidemiology Unit, Nuffield Department of Clinical Medicine, University of Oxford, Richard Doll Building, Roosevelt Drive, OX3 7LF Oxford, UK; 2 Department of Community Medicine, University of Tromsø, Tromsø, Norway; 3 Department of Preventive and Predictive Medicine, National Cancer Institute, Milan, Italy; 4 Inserm, Centre for Research in Epidemiology and Population Health, U1018, Institut Gustave Roussy, F-94805, Villejuif, France; 5 Paris South University, UMRS 1018, F-94805, Villejuif, France; 6 Institute of Cancer Epidemiology, Danish Cancer Society, Copenhagen, Denmark; 7 Department of Epidemiology, School of Public Health, Aarhus University, Aarhus, Denmark; 8 International Agency for Research on Cancer, Lyon, France; 9 Division of Cancer Epidemiology, German Cancer Research Center, Heidelberg, Germany; 10 Department of Epidemiology, German Institute of Human Nutrition, Potsdam, Germany; 11 Department of Hygiene and Epidemiology, University of Athens Medical School, Athens, Greece; 12 Hellenic Health Foundation, Athens, Greece; 13 Molecular and Nutritional Epidemiology Unit, Cancer Research and Prevention Institute, Florence, Italy; 14 Environmental Epidemiology and Cancer Registry Unit, National Cancer Institute, Milan, Italy; 15 Department of Clinical and Experimental Medicine, Federico II University, Naples, Italy; 16 Cancer Registry and Histopathology Unit, Department of Oncology, Azienda Ospedaliera ‘Civile – MP Arezzo’, Ragusa, Italy; 17 ISI Foundation, Torino, Italy; 18 Department of Epidemiology and Biostatistics, School of Public Health, Imperial College, London, UK; 19 National Institute for Public Health and the Environment, Bilthoven, The Netherlands; 20 Department of Gastroenterology and Hepatology, University Medical Centre Utrecht, Utrecht, The Netherlands; 21 Julius Center for Health Sciences and Primary Care, University Medical Center Utrecht, Utrecht, The Netherlands; 22 Public Health and Participation Directorate, Health and Health Care Services Council, Asturias, Spain; 23 Unit of Nutrition, Environment and Cancer, Cancer Epidemiology Research Programme, Catalan Institute of Oncology, Barcelona, Spain; 24 Andalusian School of Public Health and CIBER de Epidemiología y Salud Pública, Granada, Spain; 25 Department of Epidemiology, Regional Health Authority and CIBER de Epidemiología y Salud Pública, Murcia, Spain; 26 Public Health Institute of Navarra, Pamplona, Spain; 27 Public Health Division of Gipuzkoa and CIBER de Epidemiología y Salud Pública, San Sebastian, Spain; 28 Department of Oncology, Lund University Hospital, Lund, Sweden; 29 Department of Surgery, Malmö University Hospital, Malmö, Sweden; 30 Department of Medical Biosciences, Pathology, Umeå University, Umeå, Sweden; 31 Department of Public Health and Clinical Medicine, Nutritional Research, Umeå University, Umeå, Sweden; 32 Medical Research Council Dunn Human Nutrition Unit, University of Cambridge, Cambridge, UK; 33 Clinical Gerontology Unit, University of Cambridge, Cambridge, UK

**Keywords:** oral contraceptives, reproductive history, colorectal cancer, cohort study

## Abstract

**Background:**

Oral contraceptive use and reproductive factors may initiate long-term changes to the hormonal milieu and thereby, possibly influence colorectal cancer risk.

**Methods:**

We examined the association of hormonal and reproductive factors with risk of colorectal cancer among 337 802 women in the European Prospective Investigation into Cancer and Nutrition, of whom 1878 developed colorectal cancer.

**Results:**

After stratification for center and age, and adjustment for body mass index, smoking, diabetes mellitus, physical activity and alcohol consumption, ever use of oral contraceptives was marginally inversely associated with colorectal cancer risk (hazard ratio (HR), 0.92; 95% confidence interval (CI), 0.83–1.02), although this association was stronger among post-menopausal women (HR, 0.84; 95% CI: 0.74–0.95). Duration of oral contraceptive use and reproductive factors, including age at menarche, age at menopause, type of menopause, ever having an abortion, parity, age at first full-term pregnancy and breastfeeding, were not associated with colorectal cancer risk.

**Conclusion:**

Our findings provide limited support for a potential inverse association between oral contraceptives and colorectal cancer risk.

Men tend to have a slightly higher incidence of colorectal cancer than women of similar age (American Cancer Society, 2007). Oestrogen has been implicated for this decreased risk in women through mechanisms that involve reduction of secondary bile acid production (McMichael and Potter, 1980; Bayerdorffer *et al*, 1995), reduction of circulating insulin-like growth factor-I (Campagnoli *et al*, 1993; Renehan *et al*, 2004), and protection of the oestrogen receptor gene from methylation (Issa *et al*, 1994).

The epidemiologic evidence for a causal link between oral contraceptives and colorectal cancer risk is equivocal. Some studies have suggested inverse associations (Potter and McMichael, 1983; Martinez *et al*, 1997; Fernandez *et al*, 1998; Nichols *et al*, 2005; Campbell *et al*, 2007; Hannaford *et al*, 2007; Lin *et al*, 2007; Kabat *et al*, 2008), whereas others have found no association (Weiss *et al*, 1981; Bostick *et al*, 1994; Jacobs *et al*, 1994; Platz *et al*, 1997; Troisi *et al*, 1997; Levi *et al*, 2003; Purdue *et al*, 2005; Dorjgochoo *et al*, 2009; Rosenblatt *et al*, 2009). A recent meta-analysis, summarising the results from 7 cohort and 11 case–control studies, reported a statistically significant 19% reduced risk among ever users of oral contraceptives compared with never users, although there was no clear association with increasing duration of use (Bosetti *et al*, 2009). No consistent association has been observed for menstrual and reproductive variables and risk of colorectal cancer (Weiss *et al*, 1981; Potter and McMichael, 1983; Peters *et al*, 1990; Wu-Williams *et al*, 1991; Gerhardsson de Verdier and London, 1992; Bostick *et al*, 1994; Jacobs *et al*, 1994; Kampman *et al*, 1997; Martinez *et al*, 1997; Platz *et al*, 1997; Talamini *et al*, 1998; Nichols *et al*, 2005; Purdue *et al*, 2005; Lin *et al*, 2007; Sakauchi, 2007; Akhter *et al*, 2008; Kabat *et al*, 2008; Rosenblatt *et al*, 2009), although the majority of the studies are case–control or small cohort studies with low power to study dose–response associations.

We examined the associations of oral contraceptive use and reproductive variables with colorectal cancer risk in the large European Prospective Investigation into Cancer and Nutrition (EPIC) cohort.

## Materials and Methods

Study participants included 1878 female colorectal cancer cases (1295 colon and 583 rectal cancers) and 335 924 female non-cases recruited into EPIC, a prospective cohort that was established in the 1990s in 10 European countries with more than half a million participants, mostly aged 35–70 years. Incident cancer cases were identified through linkage to population cancer registries in Denmark, Italy, The Netherlands, Norway, Spain, Sweden and the UK, or with a combination of methods including linkage to health insurance records, cancer and pathology registries, and active follow-up of study participants or their next of kin in France, Germany and Greece. The colorectal cancer diagnosis was confirmed by histology for 80.2% of the cases, by clinical examination for 11.6% and the remaining 8.2% by self-report, autopsy or death certificate. Women were excluded if they had prevalent cancer at recruitment, if they did not return the baseline lifestyle questionnaire, if they never menstruated or if they had missing information on all exposure variables. Details on the cohort population, the data collection procedures and the outcome and covariate assessment methods have been described in detail elsewhere (Riboli *et al*, 2002; Tsilidis *et al*, 2010).

Women were asked at the baseline questionnaire whether they had ever used oral contraceptives, their duration of use, and age they started use. Information on age at menarche and menopause, numbers of full-term pregnancies (live and still births) and induced or spontaneous abortions, age at the first full-term pregnancy, and the reason for menopause (natural *vs* surgical) was also collected. Information on breastfeeding was collected for the first three full-term pregnancies and the last one. Menopausal status was defined according to information on menstruation status and ovariectomy, details of which are provided elsewhere (Dossus *et al.*, 2010).

Hazard ratios (HR) and their 95% confidence intervals (95% CI) for colorectal cancer were estimated using Cox proportional hazards models stratified by EPIC-participating center and age at recruitment (⩽50, 51–53, 54–56, 57–59, 60–62, 63–65, >65 years) and adjusted for smoking status (never, former and current), self-reported diabetes mellitus (no or yes), body mass index (BMI; <25, ⩾25–<30, ⩾30 kg m^−2^), physical activity (inactive, moderately inactive, moderately active, active, and alcohol intake (quartiles: <0.58, ⩾0.58–<3.61, ⩾3.61–<11.08, ⩾11.08 g per day). Missing values for smoking status (2.2%), diabetes (4.2%), physical activity (13.4%) and alcohol (0.8%) were included as a separate category in the models. An analysis that excluded women with missing values for these covariates provided very similar results, and these are not presented here. Further adjustment for menopausal hormone therapy, waist and hip circumference, waist to hip ratio, dietary variables (intakes of energy, saturated fat, fibre, folate, calcium and red meat) or mutual adjustment for oral contraceptive use and reproductive factors in relevant models gave virtually identical results. Analyses were also performed according to EPIC country, cancer subsite (colon *vs* rectum) and potentially modifying factors (age at recruitment (at the median, <51 *vs* ⩾51 years), BMI (<25 *vs* ⩾25 kg m^−2^), menopausal hormone therapy (ever *vs* never) and menopausal status (post- *vs* pre-/peri-menopausal)).

## Results

The mean ages at recruitment and diagnosis for the colorectal cancer cases were 57 and 63 years, respectively, and the mean length of follow-up in the whole cohort was 9 years. Compared with women without colorectal cancer, cases were on average older, had slightly higher BMI, drank more alcohol, exercised less and were less likely to have ever taken oral contraceptives (Table 1).

Overall, there were no statistically significant associations between oral contraceptives, reproductive factors and colorectal cancer risk (Table 2). Ever use of oral contraceptives was marginally inversely associated with risk (HR, 0.92; 95% CI: 0.83–1.02), but neither duration of use (*P*-trend, 0.41) nor age at start of use (*P*-trend, 0.32) were associated with risk. However, the association of oral contraceptive use on risk varied by menopausal status (*P*-interaction, <0.01); ever use of oral contraceptives was associated with a significantly reduced risk in post-menopausal women (HR, 0.84; 95% CI: 0.74–0.95), but no significant association was observed among pre- or peri-menopausal women (HR, 1.19; 95% CI: 0.96–1.48). There was no evidence of an interaction for duration or age at start of oral contraceptive use by menopausal status (data not shown).

Reproductive factors, including age at menarche, age at menopause, type of menopause, ever having an abortion, parity, age at first full-term pregnancy and breastfeeding, were not associated with colorectal cancer risk (Table 2). The associations between oral contraceptive use, reproductive factors and colorectal cancer risk did not differ according to country, colorectal cancer subsite and baseline characteristics (age, BMI, menopausal hormone therapy and menopausal status).

## Discussion

In this large prospective study, ever use of oral contraceptives was associated with a small reduction in colorectal cancer risk, which was stronger among post-menopausal women compared with pre-/peri-menopausal women. Although our finding of an inverse association with use of oral contraceptives is consistent with the prior literature (Bosetti *et al*, 2009), most studies have not reported a reduction in colorectal cancer risk with increasing duration of oral contraceptive use (Bosetti *et al*, 2009). This may, in part, be because of relatively small study sizes to detect a significant association, although the present study, with over 1800 cases, also found no association with duration of oral contraceptive use. Our stronger inverse finding for oral contraceptive use and colorectal cancer risk in post-menopausal women did not change after adjustment for menopausal hormone therapy and reproductive variables, and is not explained by a longer duration of oral contraceptive use among older women as there was no association between duration of use and risk overall or in subgroups by menopausal status. In addition, post-menopausal women had only a slightly longer mean duration of oral contraceptive use compared with pre-/peri-menopausal women (9.1 *vs* 8.5 years), despite being older at recruitment (58 *vs* 44 years). However, post-menopausal women were more likely to have started using oral contraceptives during the 1960 s when high-dose formulations were much more common (McMichael and Potter, 1980), which may partly explain the apparent higher risk in these women. Earlier studies have not reported significant interactions between oral contraceptive use and colorectal cancer risk by age or menopausal status (Kampman *et al*, 1997; Lin *et al*, 2007); however, one case–control study observed a non-significant reduced risk of colon cancer for ever use of oral contraceptives among women older than 62 years at recruitment, and no association among younger women (Kampman *et al*, 1997). Future studies with detailed information on the dose and hormonal constituent of the oral contraceptives are needed to clarify this association. No significant associations were found for reproductive factors, which is consistent with most of the literature (Gerhardsson de Verdier and London, 1992; Kampman *et al*, 1997; Troisi *et al*, 1997; Nichols *et al*, 2005; Lin *et al*, 2007; Sakauchi, 2007; Akhter *et al*, 2008; Kabat *et al*, 2008).

The major strength of this study is its size and power to study dose-response associations, and its detailed and standardised assessment of reproductive factors across Europe. In conclusion, oral contraceptive use was associated with a reduced risk of colorectal cancer among post-menopausal women. Duration of oral contraceptive use and reproductive factors were not associated with risk. Our findings provide limited support for a potential inverse association between oral contraceptives and colorectal cancer risk.

## Figures and Tables

**Table 1:** Participant characteristics at recruitment among women in the European Prospective Investigation into Cancer and Nutrition cohort

**Characteristic**	**Colorectal cancer cases (*n*=1878)**	**Non-cases (*n*=335 924)**
Mean (s.d.) age at recruitment (years)	57.4 (8.0)	50.5 (9.7)
Mean (s.d.) body mass index (kg m^−2^)	25.5 (4.5)	25.0 (4.5)
Mean (s.d.) alcohol intake (g per day)^a^	10 (13.7)	9.0 (12.2)
Moderately active/active (%)	31.7	34.2
Current smokers (%)	18.5	19.6
Self-reported diabetes mellitus (%)	3.0	2.3
		
*Menopausal status* (%)		
Pre-menopausal	11.2	34.2
Peri-menopausal/unknown	14.7	18.8
Post-menopausal (natural/surgical)	74.1	47.0
		
Ever oral contraceptive use (%)	43.8	58.4
Mean (s.d.) duration of oral contraceptive use (years)^b^	9.2 (9.8)	8.7 (9.3)
Mean (s.d.) age at menarche (years)	13.2 (1.6)	13.1 (1.5)
Mean (s.d.) age at menopause (years)^c^	49.1 (4.9)	48.6 (5.1)
Ever had a full-term pregnancy (%)	83.5	79.6
Mean (s.d.) number of full-term pregnancies^d^	2.4 (1.1)	2.3 (1.0)
Mean (s.d.) age at first full-term pregnancy, years^d^	25.0 (4.4)	24.8 (4.4)
Ever breastfed (%)^d^	81.2	81.8

a Among consumers only; 8.8% of the cases and 9.2% of the non-cases were non-consumers of alcohol.

b Among ever oral contraceptive users only.

c Among post-menopausal women only.

d Among women with a full-term pregnancy only.

**Table 2:** Hazard ratio (HR) and 95% confidence interval (CI) for oral contraceptive use, reproductive variables and colorectal cancer among women in the European Prospective Investigation into Cancer and Nutrition cohort

**Variable**	**Number of cases/non-cases^a^**	**Age and center-stratified, HR (95% CI)**	**Multivariable adjusted, HR (95% CI)^b^**
*Oral contraceptive use*			
Never	1040/138 359	1.00 (reference)	1.00 (reference)
Ever	822/196 040	0.93 (0.84–1.03)	0.92 (0.83–1.02)
			
*Oral contraceptive use (among post-menopausal women)*			
Never	908/89 064	1.00 (reference)	1.00 (reference)
Ever	469/67 757	0.85 (0.75–0.96)	0.84 (0.74–0.95)
			
*Oral contraceptive use (among pre-*/*peri-menopausal women)*			
Never	132/49 295	1.00 (reference)	1.00 (reference)
Ever	353/128 283	1.22 (0.99–1.51)	1.19 (0.96–1.48)
*P*-interaction		<0.01	<0.01
			
*Duration of oral contraceptive use (years)* c			
⩽1	161/36 175	1.00 (reference)	1.00 (reference)
2–4	167/42 208	0.99 (0.80–1.24)	0.99 (0.80–1.23)
5–9	150/42 409	0.94 (0.75–1.18)	0.93 (0.74–1.17)
⩾10	264/58 041	1.10 (0.90–1.36)	1.09 (0.89–1.35)
*P*-trend		0.37	0.41
			
*Age at menarche*			
<12	258/49 935	1.00 (reference)	1.00 (reference)
12	358/70 982	0.95 (0.81–1.11)	0.95 (0.81–1.12)
13	457/85 998	0.96 (0.83–1.12)	0.97 (0.84–1.14)
14	424/71 912	0.95 (0.82–1.12)	0.97 (0.83–1.14)
⩾15	352/52 954	0.95 (0.80–1.12)	0.96 (0.82–1.14)
*P*-trend		0.64	0.80
			
*Age at menopause* d			
⩽50	654/77 482	1.00 (reference)	1.00 (reference)
51–52	200/20 489	1.06 (0.90–1.25)	1.07 (0.91–1.25)
53–55	196/18 391	1.06 (0.90–1.25)	1.07 (0.91–1.26)
>55	59/5084	0.99 (0.76–1.30)	1.00 (0.76–1.31)
*P*-trend		0.58	0.54
			
*Type of menopause* d			
Natural	1303/147 920	1.00 (reference)	1.00 (reference)
Surgical	88/9 875	1.14 (0.91–1.42)	1.13 (0.91–1.41)
			
*Induced or spontaneous abortion*			
Never	1129/180 995	1.00 (reference)	1.00 (reference)
Ever	221/45 509	1.01 (0.87–1.18)	1.00 (0.86–1.17)
			
*Full-term pregnancy*			
Never	231/49 342	1.00 (reference)	1.00 (reference)
Ever	1568/267 467	0.96 (0.83–1.10)	0.96 (0.83–1.10)
			
*Number of full-term pregnancies* e			
1	250/49 177	1.00 (reference)	1.00 (reference)
2	721/129 409	1.16 (1.00–1.34))	1.16 (1.01–1.35)
3	384/61 490	1.15 (0.98–1.36)	1.16 (0.98–1.36)
⩾4	213/27 391	1.17 (0.97–1.41)	1.17 (0.97–1.42)
*P*-trend		0.15	0.15
			
*Age at first full-term pregnancy* e			
⩽20	210/40 007	1.00 (reference)	1.00 (reference)
21–23	433/71 912	1.01 (0.86–1.19)	1.02 (0.86–1.21)
24–25	320/52 140	0.97 (0.81–1.16)	0.98 (0.82–1.18)
26–30	439/75 488	0.90 (0.76–1.07)	0.92 (0.77–1.09)
>30	163/26 886	0.98 (0.79–1.20)	0.99 (0.80–1.22)
*P*-trend		0.22	0.30
			
*Breastfeeding* e			
Never	211/39 471	1.00 (reference)	1.00 (reference)
Ever	1273/218 695	1.12 (0.97–1.31)	1.13 (0.97–1.32)

a The number of cases and non-cases do not add up to the total number of 1878 cases and 335 924 non-cases because of missing values.

b From a Cox proportional hazards model stratified by the European Prospective Investigation into Cancer and Nutrition participating center and age at recruitment, and adjusted for smoking status (never, former, current), diabetes mellitus (never, ever), body mass index (<25, ⩾25–<30, ⩾30 kg m^−2^), physical activity (inactive, moderately inactive, moderately active, active), and alcohol use (<0.58, ⩾0.58–<3.61, ⩾3.61–<11.08, ⩾11.08 g per day).

c Among ever oral contraceptive users only.

d Among post-menopausal women only.

e Among women with a full-term pregnancy.
